# Thiazolidin-4-Ones as a Promising Scaffold in the Development of Antibiofilm Agents—A Review

**DOI:** 10.3390/ijms25010325

**Published:** 2023-12-26

**Authors:** Nazar Trotsko

**Affiliations:** Department of Organic Chemistry, Faculty of Pharmacy, Medical University of Lublin, 4A Chodźki Street, 20-093 Lublin, Poland; nazar.trotsko@umlub.pl

**Keywords:** thiazolidin-4-one, rhodamine, thiazolidine-2,4-dione, biofilm, antibiofilm activity

## Abstract

Thiazolidin-4-ones have a broad range of medical and clinical implementation, which is important for pharmaceutical and medicinal chemistry. This heterocyclic core has been reported to possess a diversity of bioactivities, including antimicrobial and antibiofilm-forming potential. The resistance of biofilms to antibiotics or disinfectants is a serious medical problem. Therefore, there is a natural need to discover new effective structures with properties that inhibit biofilm formation. This review aims to analyze the antibiofilm features of thiazolidin-4-ones described in the literature over the last two decades. The information gathered in this review could benefit the rational design of new effective antibiofilm small molecules with thiazolidin-4-one cores.

## 1. Introduction

The effectiveness of antimicrobial therapy faces a significant challenge in the form of resistance development, observed in both bacteria and fungi. This resistance is a strategic adaptation for self-survival. It is now known that more than 99% of bacteria in the natural environment exist in the form of biofilm and not as single planktonic cells. The resistance of bacterial biofilms to antibiotics or commonly used disinfectants is a serious problem in modern medicine and industrial practice. Currently, it is estimated that biofilm-mediated infections account for approximately 80% of all infections affecting animals and humans [1,2]. Microorganisms that adhere to the surface form durable, thin films called biological membranes or biofilms. Currently, biofilms are defined as complex multicellular structures of bacteria surrounded by a layer of organic and inorganic substances produced by these microorganisms, showing adhesion to both biological and abiotic surfaces. Microbial cells, which are part of the biofilm, are nearly 1000 times more resistant to the effects of toxic substances (disinfectants, antibiotics, and surfactants) than those remaining in suspension [3,4]. Living in clusters, microorganisms develop multi-level defense mechanisms against the degrading effects of chemotherapy drugs on cells.

The role of biofilms in antimicrobial resistance is highly complex and may significantly drive resistance. As an illustration, consider a study that investigated the antibiotic resistance of *Staphylococcus epidermidis* within biofilms. In the planktonic state, all isolates demonstrated susceptibility to the antibiotic vancomycin, reaching 100%. However, when tested within a biofilm, almost 75% of these isolates exhibited complete resistance to the same antibiotic [5].

Bacteria commonly employ antibiotic resistance mechanisms such as point mutations, enzymes, and efflux pumps [6,7,8]. However, these mechanisms are less likely to account for the resistance observed in biofilm organisms. Within a biofilm, various components collaboratively act to diminish or completely impede antibiotic effectiveness, actively contributing to the reinforcement of resistance. When these mechanisms operate in concert, they enable the survival of organisms within the biofilm even in the presence of elevated antibiotic concentrations, a phenomenon referred to as resistance.

One of the directions to combat biofilm is the use of new small molecules with confirmed biological potency, especially antimicrobial and/or antibiofilm properties. Such a group of derivatives may be compounds with a thiazolidin-4-one core. Thiazolidin-4-one derivatives exhibit a broad spectrum of therapeutic applications and hold clinical significance in the field of medicinal chemistry [9,10,11,12,13]. For example, thiazolidine-2,4-diones are a well-known group of antidiabetic drugs, glitazones, that act by activating PPARγ. Representants of this group, pioglitazones and rosiglitazones, are still present in the pharmaceutical market in the EU and the US. Another thiazolidine-4-one derivative, the drug ponesimod, is used for the treatment of multiple sclerosis. It was approved for medical use in the US and EU in 2021. It is a sphingosine-1-phosphate receptor modulator [14,15]. Moreover, the compounds with a thiazolidin-4-one core have been documented to display diverse biological activities such as antibacterial [16,17,18], antifungal [19], antitubercular [20], anticancer [21,22], antiviral [23], and antiprotozoal activities [24].

Previous information about the antibiofilm activity of thiazolidin-4-ones was unsystematic and generally found in reviews about the biological potential of thiazolidin-4-ones [25]. Nevertheless, over the last two decades, various thiazolidin-4-one derivatives have been tested for their antibiofilm properties, and some of them have demonstrated promising results.

The main assumption of this study is to collect, systemize, and analyze information about the antibiofilm potential of thiazolidin-4-one derivatives reported in the scientific literature between the years 2004 and 2023. This review describes thiazolidin-4-ones, which have antibiofilm activity against different bacteria and fungi by inhibiting their formation or interfering with their construction, leading to the removal of existing biofilm.

## 2. Biofilm Life-Cycle

**Figure 1:**
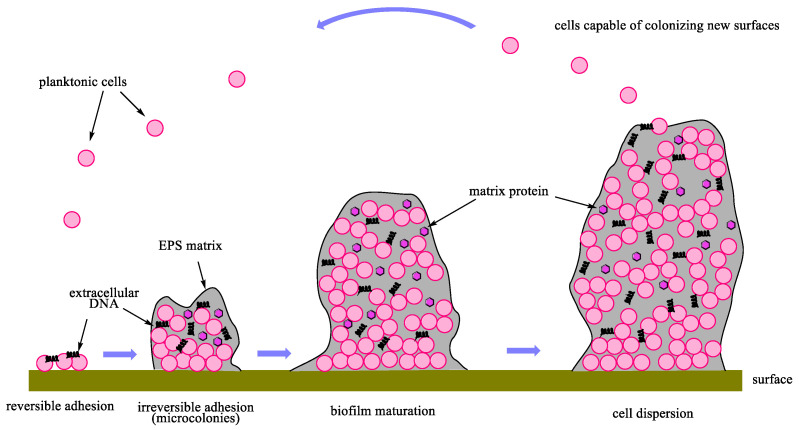
A typical biofilm life cycle.

The formation of a biofilm is a multi-stage process that depends on the structure and properties of the colonized surface, as well as on the properties of the microorganisms. In the first stage, the bacteria settle in planktonic form and attach themselves to the substrate. Cells bind to the medium by nonspecific, reversible interactions. In the second step, a specific reaction takes place between the bacterial adhesins and the substrate. The close adherence of the microbial cell to the substrate for a sufficiently long time results in an irreversible connection. In this phase, microorganisms produce an extracellular polymeric substance (EPS), which becomes the extracellular matrix. In the next stage, the multiplication and differentiation of microorganisms are observed. In the final stage of biofilm formation, bacterial cells detach from the formed structure and, because of moving to new surfaces, give rise to a new biofilm (Figure 1) [26].

## 3. Strategies to Control Harmful Biofilms

Current strategies to control harmful biofilms may be classified into three categories: (i) changing abiotic surface characteristics to prevent biofilm formation; (ii) regulating the signaling pathways to inhibit biofilm formation and stimulate biofilm dispersal; (iii) applying external forces to eradicate the biofilm [27]. The first category is changing abiotic surfaces including by treating abiotic surfaces and coating surfaces. Biofilm development is dependent on the physicochemical features of adsorptive surfaces. Consequently, altering the properties of material surfaces, including smoothness, wettability, or hydrophilicity, holds the potential to put a stop to the formation of biofilm. In turn, the coating of small molecules can also change the adhesive properties of surface materials. For example, coating tissue implants and medical devices can decrease microbial adhesion to the material surface to prevent biofilm formation and reduce bacterial infection.

The next category is the regulation of signaling pathways, such as quorum sensing (QS), leading to the inhibition of biofilm development. QS is a phenomenon of chemical “communication” of microorganisms consisting of the production and secretion of signal molecules (autoinducers) into the environment, which are used in various physiological processes, including in the formation of biofilm. The increase in the concentration of autoinductors (AIs) is a function of the number of cells. QS systems typically comprise an enzyme responsible for synthesizing the signaling molecules (e.g., acyl-homoserine lactones (AHL) or cyclic peptides) and a receptor that binds these signals, triggering the reprogramming of gene expression. This process includes the activation of genes, including those encoding the enzyme responsible for signal production, establishing a positive feedback loop. In bacterial pathogens, most QS-controlled genes codify diverse virulence factors, such as proteases, toxins, and adhesins. Shortly, QS regulates the metabolic activity of planktonic cells, and it can induce microbial biofilm formation and increased virulence [28].

Mechanisms of the QS inhibiting agents in controlling bacterial biofilm formation may include the next important points: (i) inhibition of AI synthesis; (ii) degradation or inactivation of AIs by AHL-lactonases, -oxidoreductases, and antibodies; (iii) interference in the signal receptors using AI antagonists; (iv) interference in the response regulators, thus disturbing signaling cascade; (v) reducing the extracellular AI accumulation by inhibiting AI efflux, thus inhibited cell-to-cell signaling [29].

Microorganisms’ communication can occur between cells of one species or of different species. The chemical nature of the signals, their mechanisms of action, and the genes that control quorum sensing vary from case to case. Regulation of biofilm formation affords a novel potential target to control biofilms [26]. Other biofilm inhibitors are based on nucleotide second messenger molecules. These molecules play an important role in regulating the various physiological functions of bacteria [30]. The last category is the use of external forces for biofilm eradication, such as physical methods (ultrasound and magnetic fields) and biochemical methods (application of phage lysins and degradative enzymes).

## 4. Thiazolidin-4-Ones with Antibiofilm Properties

### 4.1. 2-Iminothiazolidin-4-Ones

**Figure 2:**
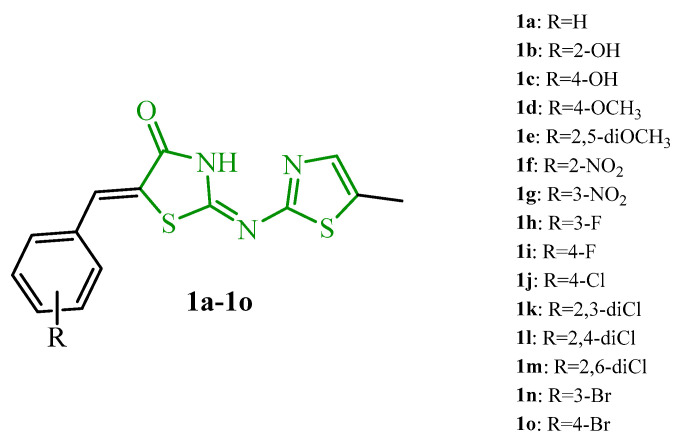
The structures of thiazolidin-4-one-thiazole hybrids.

Haroun et al. [31] obtained a series of 4-thiazolidinone-thiazole hybrids (green color in Figure 2). The series of these derivatives showed antimicrobial activity with MIC in the range of 26.3 to 378.5 µM. Among them, the five most active derivatives (**1b**, **1g**, **1h**, **1m**, and **1n**) were tested for inhibition of *P. aeruginosa* biofilm formation. The percentage of reduction for **1h**, **1m**, and **1n** was above 50% (61.34%, 62.69%, and 56.74%, respectively), recorded after application of concentrations equal to their MIC (125.4 µM, 162.1 µM, and 157.9 µM, respectively), indicating good biofilm-inhibiting potential. It is worth noticing that compounds with good antibiofilm potential had 3-F, 3-Br, or 2,6-diCl substituents in the benzylidene fragment of the molecule.

**Figure 3:**
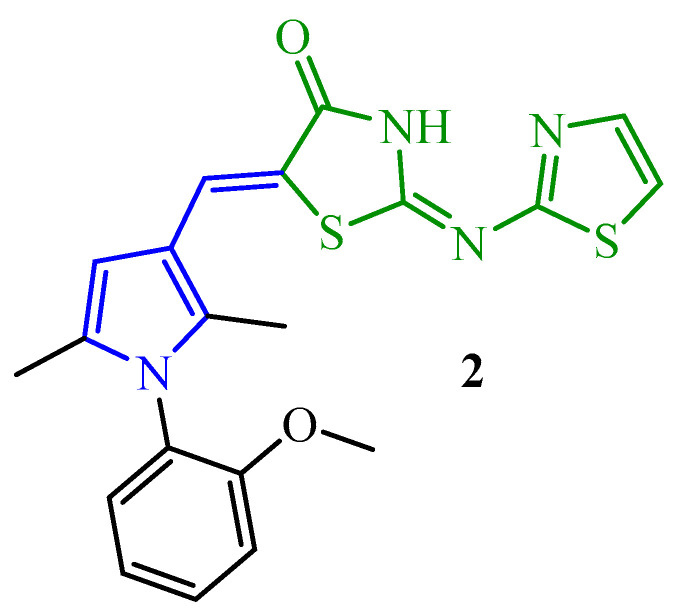
The structure of pyrrole-thiazolidin-4-one hybrid with antibiofilm activity. Green color—highlighted the 4-thiazolidinone-thiazole core.

The introduction at the fifth position of thiazolidine ring pyrrolemethylidene moiety (blue color in Figure 3) allowed the obtaining of the effective compound **2** against *S. aureus* (MIC = 0.5 µg/mL). Therefore, this compound was tested on its antibiofilm activity. As shown in the results, compound **2** reduced *S. aureus* biofilm by >11% at 10× MIC. This result was compared to standard drugs such as levofloxacin and vancomycin [32].

**Figure 4:**
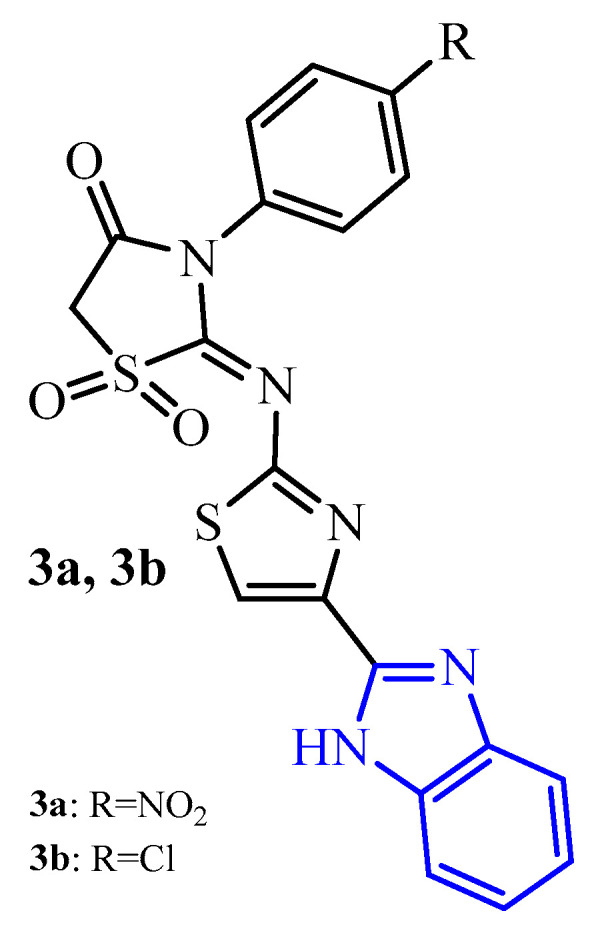
The structures of thiazolidin-4-one-thiazol hybrids with benzimidazole moiety (blue color).

Gullapelli and Maroju [33] synthesized a series of iminothiazolidin-4-ones and evaluated their antibacterial and antibiofilm potential. Compounds **3a** and **3b** showed good antibacterial potential against four strains including two resistants with MIC in the range of 3.62 to 7.14 µg/mL for compound **3a** and with MIC in the range of 2.95 to 4.63 µg/mL for compound **3b**. The antibiofilm activity of compounds **3a** and **3b** was very promising (Figure 4). Compound **3a** showed antibiofilm activity against resistant strains MRSA and VRE with biofilm inhibitory concentrations (BICs) of 8.23 µg/mL and 7.56 µg/mL, respectively. Moreover, derivative **3a** exhibited activity against *K. pneumoniae* and *E. coli* biofilms with BICs of 6.25 and 6.62 µg/mL, respectively. Significant activity against biofilm formation of the above strains showed also in compound **3b**. Their BICs were 2.22 µg/mL against MRSA and 3.05 µg/mL against VRE strains. In addition, **3b** was found to be significant against *K. pneumoniae* and *E. coli* biofilms with BICs of 3.25 and 2.03 µg/mL, respectively.

**Figure 5:**
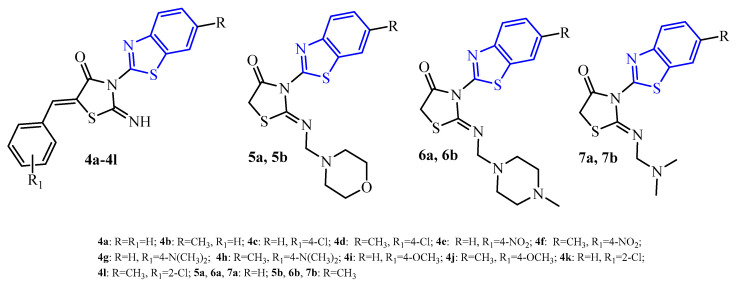
The structures of a series of 2-iminothiazolidin-4-one derivatives with benzothiazole substituent.

The Hemeda research group [34] synthesized two series of 2-iminothiazolidin-4-one derivatives with benzothiazole substituent (blue color in Figure 5) in position 3 of the thiazolidine ring (5-arylidene derivatives (**4a**–**4l**) and Mannich bases (**5a**, **5b**, **6a**, **6b**, **7a**, and **7b**)). These compounds were evaluated for their *C. albicans* antibiofilm activity. Only 10 compounds (**4a**–**4d**, **4h**, **4j**, **5b**, **6b**, **7a**, and **7b**) that showed MICs in the range of 25 to 100 µg/mL in antimicrobial screening were studied for their antibiofilm activity. Most of the tested antifungal agents against two pathogenic *Candida* isolates, CA1 and CA2, demonstrated significant antibiofilm activity compared to the reference, fluconazole, but were not more effective. Compound **4j** with the *para*-methoxy group in the benzylidene substituent showed the most antibiofilm activity against both CA1 and CA2 isolates (OD_570mm_ = 0.297 and 0.218, respectively). The Mannich base **7b** with a dimethylamino substituent in position 2 of the thiazolidine ring showed almost similar OD (OD_570mm_ = 0.266) to compound **4j** (MIC = 25 µg/mL) against only the CA2 pathogen, although its MIC value was 100 μg/mL. On the other hand, a lack of a substituent (compounds **4a** and **4b**) or 4-dimethylamino group (compound **4h**) in the benzylidene fragment allows decreasing antibiofilm activity. Their OD_570mm_ was in the range of 0.779 to 1.269. The remaining compounds (**4c**, **4d**, **5b**, **6b**, and **7a**) showed good to moderate antibiofilm activity against both isolates (OD_570mm_ were in the range 0.307 to 0.530) comparing to fluconazole (OD_570mm_ = 0.088 and 0.105 for CA1 and CA2, respectively).

**Figure 6:**
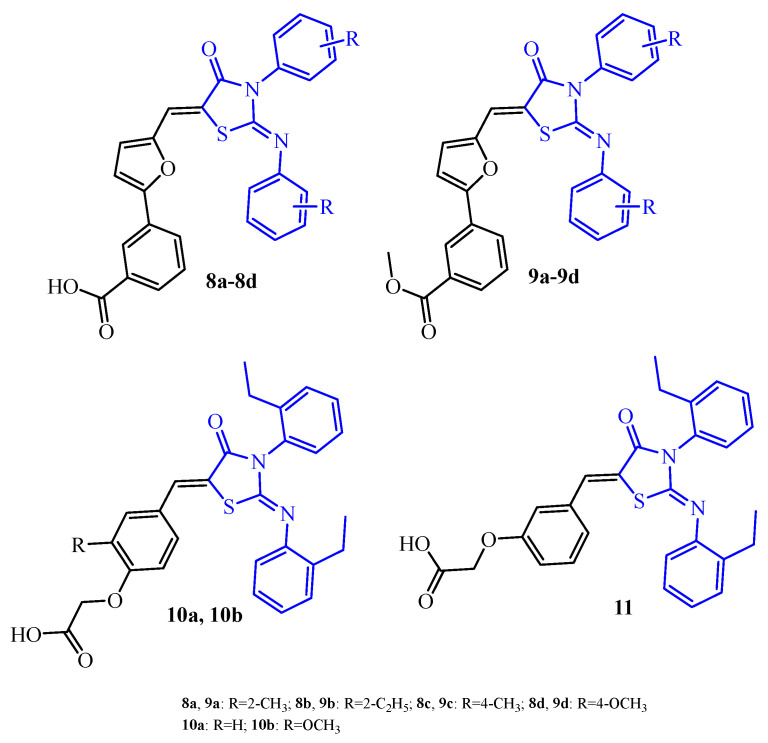
The structures of 2-iminothiazolidin-4-ones **8a**–**11**.

Pan et al. [35] designed and synthesized a series of 2-arylimino-3-arylthiazolidin-4-ones (**8a**–**11**) (blue color in Figure 6). All 25 compounds of this series were evaluated for their antibacterial and antibiofilm activity against *S. epidermidis* RP62A using a standard tube-dilution assay. Recently, some thiazolidin-4-one derivatives were documented with moderate antibiofilm activity against *S. epidermidis* RP62A, which caused their effect through inhibition of the YycG histidine kinase [36]. YycG plays an essential role in cell viability and related cell wall metabolism, biofilm formation, virulence, and antibiotic resistance. For these reasons, YycG histidine kinase may be considered as a potential target for this series of compounds.

The eighteen derivatives of this series showed weak antibacterial effects and antibiofilm activity. Their MIC values were >200 µM, and antibiofilm concentration was more than 200 µM. Compounds **10a**, **10b**, and **11** exhibited a little better antibacterial and antibiofilm activity. MIC and antibiofilm concentrations were 50 and 100 µM, respectively. The most active group of compounds in this series were derivatives **8a**–**8d** with MIC values in the range of 6.25 to 12.5 µM and antibiofilm concentration in the range of 6.25 to 25 µM. Compound **8d** with the 4-methoxy group in the benzene ring showed the best activity in the series with MIC and an antibiofilm concentration of 6.25 µM. It is worth noticing that replacement of the carboxylic group in the **8a**–**8d** derivatives by the methoxycarbonyl group (**9a**–**9d**) led to abolished antibacterial (MIC > 200 µM) and antibiofilm activity (antibiofilm concentration > 200 µM).

**Figure 7:**
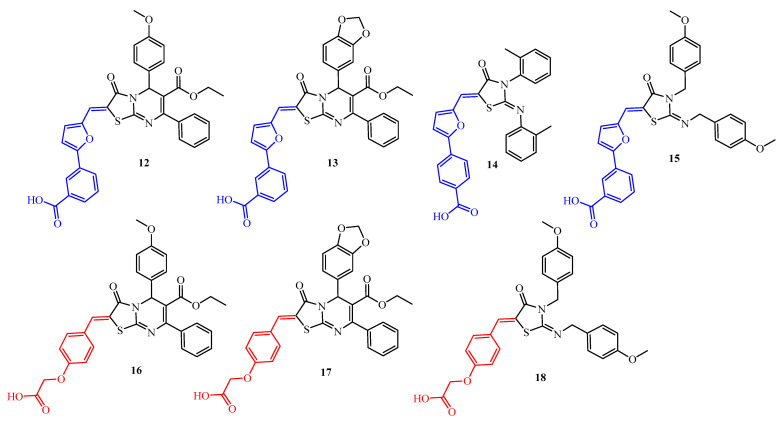
The structures of 2-iminothiazolidin-4-ones **12**–**18**.

Continuing their research, Pan et al. [37] focused on optimizing the structure with documented antibiofilm activity (compound **13**) by different pathways of modification: functional group modification, ring opening, chain shortening, and pharmacophore overlapping (Figure 7). The antibacterial and antibiofilm activity was determined using the *S. epidermidis* ATCC 35984 strain. The structure–activity relationship analysis was carried out. Compound **13** is a pioneering antibiofilm agent that has moderate inhibition of *S. epidermidis* biofilm growth (antibiofilm concentration was 25 µM). Its antibiofilm effect is associated with the inhibition of the YycG histidine kinase [36]. The modification of functional groups did not improve antibacterial and antibiofilm activities. Only the *para*-methoxyphenyl derivative (**12**) displayed antibiofilm and antibacterial activities comparable to compound **13**. In contrast, pharmacophore overlapping modification (compounds **16** and **17**) is a less potent agent than compound **13** with an antibiofilm concentration of 50 µM and MIC = 25 µM. This confirms that the furanylbenzoic acid fragment is more significant than the 2-phenoxyacetic acid substituent (blue and red colors in Figure 7, respectively). Interestingly, that ring opening led to compound **18**, which was more active against biofilm growth than derivative **16**. Unexpectedly, modification by chain shortening (**8a**, **8c**, **8d**, and **14** derivatives) led to the improvement of antibacterial and antibiofilm activities. Compound **8d** showed eightfold better antibiofilm activity than their 2,3-di(4-methoxyphenyl)methyl analogue (**15**), whereas compound **14** was fourfold more active relative to biofilm formation than derivative **13** (antibiofilm concentration value was 6.25 µM).

These results showed that the presence of a 4-(5-ylidenemethylfuran-2-yl)benzoic acid fragment and substituted phenyl moiety, which directly connected with the *N* atom of the thiazolidine ring, might be two decisive factors that advantageously influenced the antibiofilm activity.

Continuing research on the antibiofilm activity of 2-iminothiazolidin-4-one derivatives against the *S. epidermidis* ATCC 35984 strain, Huang et al. [38] synthesized six alkylphenyl analogues (**8a**–**8d**, **10a**, and **14**—structures seen in Figure 6 and Figure 7, respectively) of the YycG inhibitor compound **10b**. This study evaluated inhibition of YycG’s enzyme activity and the effects of derivatives on *S. epidermidis* biofilm proliferation. The group of 3-(5-ylidenemethylfuran-2-yl)benzoic acid (**8a**–**8d**) and 4-(5-ylidenemethylfuran-2-yl)benzoic acid (**14**) derivatives showed good antibiofilm activity with MBKC in the range of 3.1 to 13.1 µg/mL that was from 4- to 16-fold better than MBKC for compound **10b**. The most active compounds were **8d** and **14** with MBKC 3.3 and 3.1 µg/mL, respectively. Moreover, compounds from the group of 3-(5-ylidenemethylfuran-2-yl)benzoic acid derivatives **8a** and **8c** demonstrated YycG’s enzyme inhibition activity in concentrations of 34.83 µM (17.2 µg/mL) and 22.15 µM (10.9 µg/mL), respectively. These IC_50_ values were better compared to compound **10b** (IC_50_ = 47.9 µM (24.9 µg/mL)).

**Figure 8:**
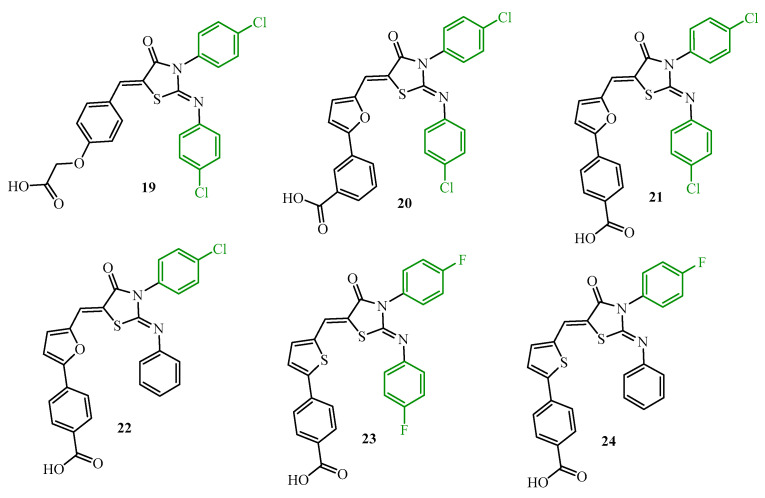
The structures of halogenphenyl 2-iminothiazolidin-4-ones.

The next step for the continuation of research on the antibiofilm potential of 2-iminothiazolidin-4-one derivatives was a synthesis of halogenphenyl analogues (**19**–**24**) of compounds **8a**–**8d**, **10a**, and **14** (green color in Figure 8). This paper evaluated the inhibition effects of new derivatives on the autophosphorylation activity of the purified recombinant YycG’s activity on *S. epidermidis* biofilm, as well as their antibacterial efficacy in a rabbit subcutaneous *S. epidermidis* biofilm infection model [39]. The IC_50_ values of derivatives **19**–**24** toward YycG’s protein were in the range of 24.2 to 71.2 µM. The most active was compound **24** (IC_50_ = 24.2 µM), which demonstrated a twofold lower concentration than precursor **10b** (IC_50_ = 47.9 µM). Furthermore, compound **24** was most effective against *S. epidermidis* biofilms. Their MBEC was 6.3 µM. Other derivatives (**20**–**23**), except **19** (MBEC = 50 µM), showed antibiofilm activity with MBEC = 12.5 µM, which was eight-fold better than precursor **10b** (MBEC = 100 µM). It is worth noticing that the incorporation of a furan (**20**–**22**) and thiophene (**23** and **24**) ring and the introduction of halogen into the appropriate position of phenyl substituents may improve antibacterial and antibiofilm activities.

Additionally, two compounds, **19** and **24,** were tested in vivo using a rabbit subcutaneous *S. epidermidis* biofilm infection model. Bacterial viability after being treated by **19** and **24** was significantly reduced compared to controls (DMSO and vancomycin 5.3 log_10_CFU/cm^2^ and 5.21 log_10_CFU/cm^2^, respectively). CFUs in biofilms treated with **19** were reduced to 2.91 log_10_CFU/cm^2^ and with **24** to 2.18 log_10_CFU/cm^2^. This is in accordance with antibiofilm activity in vitro.

Compounds **20** and **24** with antibiofilm activity against *S. epidermidis* ATCC 35984 (MBEC values were 12.5 µM and 6.3 µM, respectively) were evaluated on their antibiofilm potential against clinical staphylococcal strains [40]. Confocal microscopy with live/dead staining exhibited that at a concentration of 4× MIC (6.25 µM) of compounds **20** and **24** had bactericidal activity against six strong biofilm-forming clinical isolates (MRSA 1000234, MRSA 916054, MSSA 1815, MRSE 1020081, MRSE 915296, and MSSE 914111). The percentages of viable cells in the biofilms of six strains were 3.5% to 16.2% after compound **20** was treated. In the case of compound **24**, the percentage of viable cells in the biofilms was 1.6% to 11.6%. For the controls vancomycin and DMSO, 75.4–80.8% and 88.5–93.6% of the cells were viable in the biofilm, respectively.

**Figure 9:**
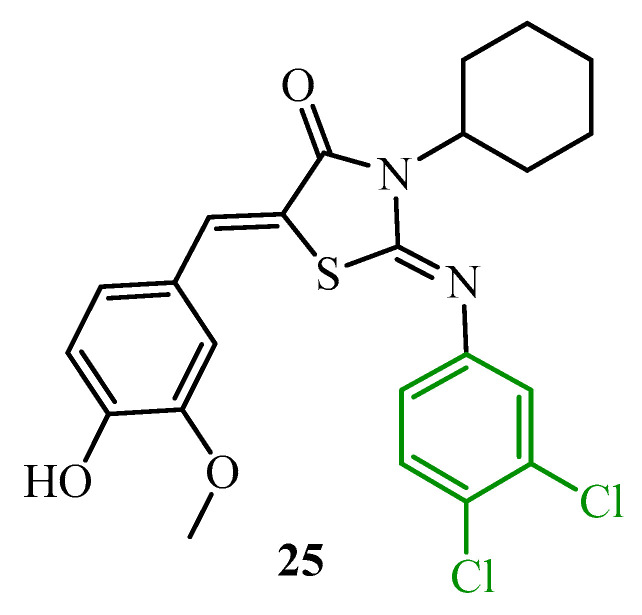
The structure of 3-cyclohexyl-2-[(3,4-dichlorophenyl)imino]-5-(4-hydroxy-3-methoxybenzylidene) thiazolidin-4-one.

Hammad and co-workers carried out the synthesis and antimicrobial evaluation of two series of halogenated thiazolidin-4-ones against clinically relevant bacterial strains. The most effective, compound **25** (MIC = 32 µg/mL against methicillin-resistant *Staphylococcus aureus* NRS384 (MRSA USA300)), was tested for its antibiofilm activity against the above-mentioned staphylococcal strain (Figure 9). The test evaluated the percentage of eradication of MRSA USA300 mature biofilm in relation to DMSO. Compound **25**, at 2× MIC, disrupted about 17% of the MRSA USA300 biofilm mass, which was better in biofilm eradication with comparison to the reference, vancomycin (which disrupted about 12% of the biofilm mass) [41].

These results [39,40,41,42] confirm the importance of the halogenphenyl substituent (green color in Figure 8 and Figure 9) in the thiazolidine ring for the antibiofilm activity among 2-iminothiazolidin-4-ones.

**Figure 10:**
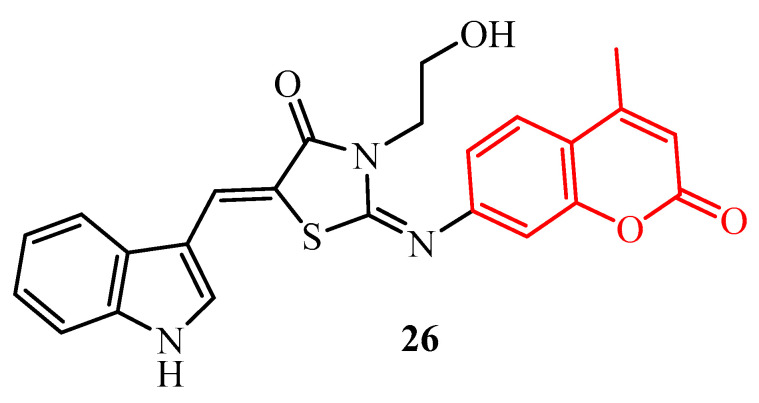
The structure of 5-[(1*H*-indol-3-yl)methylidene]-3-(2-hydroxyethyl)-2-[(4-methyl-2-oxo-2*H*-chromen-7-yl)imino]thiazolidin-4-one.

Another 2-iminothiazolidin-4-one derivative (**26**) conjugated with coumarin moiety (red color in Figure 10) showed high activity against Gram-negative bacteria *Acinetobacter baumannii* (MIC = 0.25 µg/mL). Viability of *A. baumannii* biofilm was decreased to 45% at a concentration of 2 µg/mL (8× MIC) [43].

**Figure 11:**
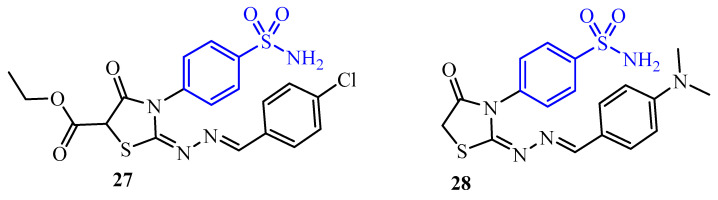
The structures of 2-(benzylidenehydrazono)thiazolidin-4-ones with sulfonamide group.

El-Hossary et al. synthesized a series of 2-(benzylidenehydrazono)thiazolidin-4-ones with a sulfonamide group in the third position of the thiazolidine ring (blue color in Figure 11). Among this series of derivatives, only two compounds (**27** and **28**) showed 70% and 80% inhibition of the biofilm formation in *S. epidermidis* at a concentration of 40 µM, respectively (Figure 11). Analyzing the substitution at the thiazolidine ring, the authors concluded that the presence of an ethylcarboxylate fragment at position 5 was not beneficial for antibacterial and biofilm inhibition activity [44].

### 4.2. 2-Arylthiazolidin-4-Ones

**Figure 12:**
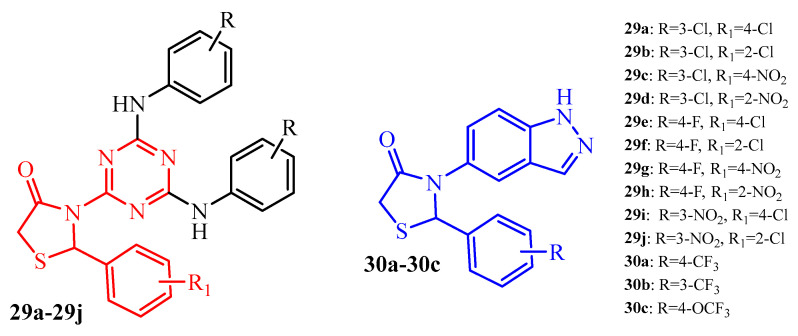
The structures of 2-arylthiazolidin-4-ones with triazine and indazole moieties.

Cystic fibrosis (CF) is a genetic disorder that affects mostly the lungs. The most common organisms causing lung infections in CF patients are *S. aureus*, *H. influenzae*, and *P. aeruginosa*. Srivastava et al. [45] synthesized a series of thiazolidin-4-one-1,3,5-triazine hybrids (**29a**–**29j**) (red color in Figure 12). These compounds were evaluated for their antibiofilm activity against *S. aureus* NCIM 2079 and *P. aeruginosa* NCIM 2036 strains. The compounds **29a**, **29c**, **29d**, **29f**, and **29g** showed moderate antibiofilm activity against *S. aureus*. Their MIC values, except **29f** (MIC = 31.25 µg/mL), were 62.5 µg/mL vs. 3.91 µg/mL for standard vancomycin. Other thiazolidin-4-one-1,3,5-triazine hybrids exhibited mild antibiofilm activity (**29b**, **29h**, and **29j** with MIC = 125 µg/mL) or no activity (**29e** and **29i**). In the case of *P. aeruginosa*, compounds **29b** and **29c** displayed no activity. It is worth noticing that compound **29f** was the most active among other derivatives, and its MIC (7.81 µg/mL) was comparable to the reference, vancomycin. Other analogues exhibited moderate activity against *P. aeruginosa* with MIC in the range of 31.25 to 62.5 µg/mL.

New series of indazole-thiazolidin-4-one hybrids were obtained and evaluated for their antibacterial and antibiofilm activity (blue color in Figure 12) [46]. *Klebsiela planticola* MTCC 530 is an important nosocomial pathogen causing urinary tract infections. For this reason, compounds **30a**–**30c** with promising bactericidal activity against *K. planticola* (MIC = 3.9 µg/mL and MBC = 15.6 µg/mL) were tested for specific *K. planticola* antibiofilm activity. These hybrids showed promising antibiofilm activity with IC_50_ values in the range of 20.28 to 20.79 µg/mL [46].

**Figure 13:**
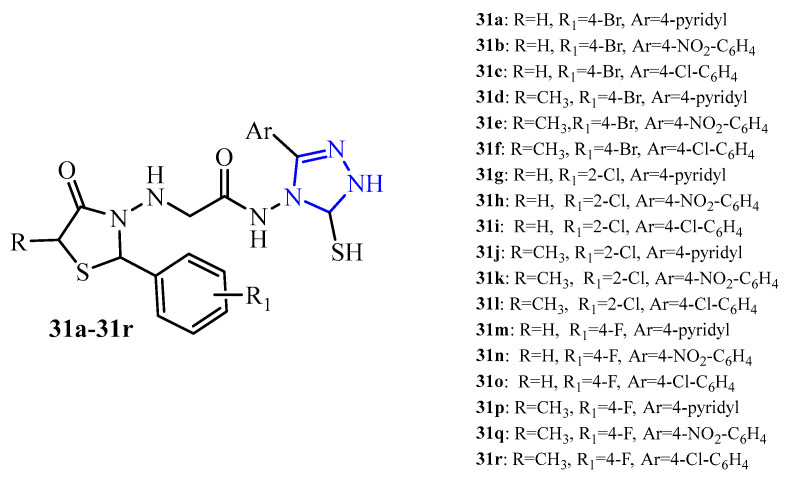
The structures of 2-arylthiazolidin-4-one-1,2,4-triazole hybrids.

A series of 2-aryl-3-aminothiazolidin-4-one derivatives with 1,2,4-triazole moiety (**31a**–**31r**) (blue color in Figure 13) were synthesized and studied for antibacterial and antibiofilm activity. Screening results showed that derivatives inhibited the *S. aureus* biofilm formation with IC_50_ in the range of 25 to 100 µg/mL. There is a mild to moderate effect. Hybrid **31n** with 4-nitrophenyl substituent in the 1,2,4-triazole ring and 4-fluorophenyl in position 2 of the thiazolidine ring was most effective in this series. Its IC_50_ was 12.5 µg/mL, which was a higher potential than the standard ciprofloxacin (IC_50_ = 25 µg/mL) [47].

**Figure 14:**
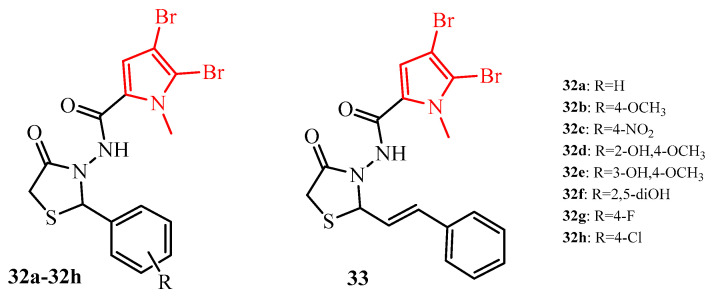
The structures of 2-arylthiazolidin-4-ones with 2,3-dibromopyrrole substituent.

Another series of 2-aryl-3-aminothiazolidin-4-one derivatives with 2,3-dibromopyrrole substituent (red color in Figure 14) (**32a**–**32h**, and **33**) showed very promising antibiofilm potential (Figure 14). The compounds of these series exhibited inhibition of *S. aureus* biofilm at a concentration in the range of 0.78 to 6.25 µg/mL; *S. epidermidis* biofilm with MIC = 3.125–12.5 µg/mL and *E. faecalis* biofilm was inhibited at a concentration in the range of 6.25–12.5 µg/mL. The compounds **32b**–**32h** exhibited equally *S. epidermidis* antibiofilm activity comparable to reference vancomycin (MIC = 3.125 µg/mL). It is worth noticing that compounds **32b** and **32c** showed antibiofilm activity against *S. aureus* with MIC = 0.78 µg/mL, and compounds **32d**, **32f**, **32g**, and **32h** with MIC = 1.56 µg/mL, which speaks to their potential in the development of newer antibiofilm agents [48].

In the context of SAR analysis, it is also worth noting that the replacement of the 2-imino group of compounds **4a**–**4l** (with benzothiazole substituent at the third position of the thiazolidine ring (blue color in Figure 5)) by 2-aryl substituent completely abolished antibiofilm activity [49].

### 4.3. 2-Thioxothiazolidin-4-Ones (Rhodanines)

Another chemical group of thiazolidin-4-ones that exhibited antibiofilm activity are the rhodanines (2-thioxothiazolidin-4-ones).

**Figure 15:**
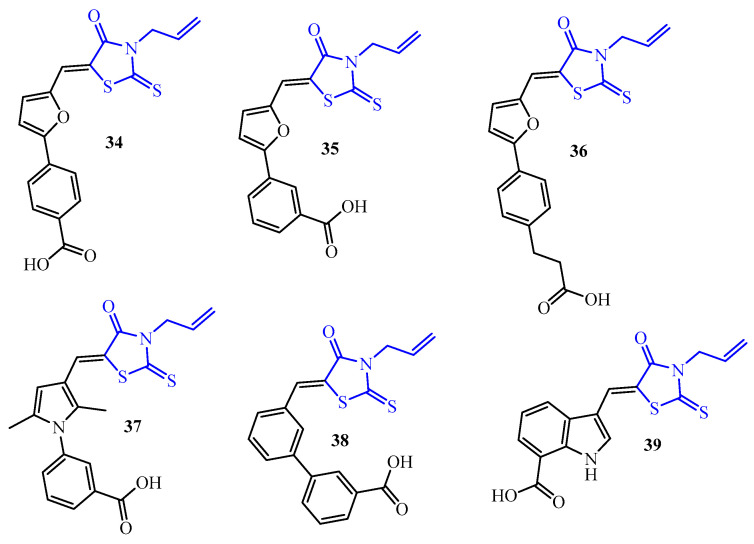
The structures of 3-allylrhodanines (blue color).

Gualtieri et al. reported antibiofilm activity of rhodanine derivative **34**, namely 4-(5-{[4-oxo-3-allyl-2-thioxo-1,3-thiazolidin-5-ylidene]methyl}furan-2-yl)benzoic acid (Figure 15) [50]. This compound is a *para*-isomer of 3-(5-{[4-oxo-3-allyl-2-thioxo-1,3-thiazolidin-5-ylidene]methyl}furan-2-yl)benzoic acid (**35**) that was selected from the group of rhodanine derivatives with strong bactericidal properties and low toxicity [51]. Compound **34** as **35** and other representatives of this group showed their antibacterial activity by inhibition of bacterial RNA polymerase. In this study, the authors compare the activity of rifampicin with that of compound **34** and reference antibiotics on *S. epidermidis* biofilms (reference *S. epidermidis* ATCC 35984 biofilm and clinical isolates *S. epidermidis* DSM 3269, *S. epidermidis* 40004, *S. epidermidis* 48155). Compound **34** demonstrated a similar potential of biofilm inhibition to rifampicin against *S. epidermidis* (ATCC 35984) biofilm at a concentration of 20 µg/mL (3.9 ± 0.083 vs. 3.48 ± 0.087 log_10_cfu/mL). Moreover, **34** showed similar levels of activity at a concentration of 20 µg/mL toward three *S. epidermidis* clinical isolates: 4 ± 0.239 (DSM 3269), 3.8 ± 0.055 (40004), and 3.4 ± 0.032 (48155) log_10_cfu/mL [50]. As a continuation of these studies, a series of phenyl-furanyl-rhodanines and their analogues as inhibitors of RNA polymerase were synthesized and evaluated for their antibacterial and antibiofilm activity. Among the tested compounds, five derivatives displayed good activities on *S. epidermidis* biofilm at a concentration of eightfold the MIC (MIC = 25 µg/mL for **36**–**39** and 12.5 µg/mL for **35**) and a decreased amount of bacteria by 2–3 log_10_cfu/well. It is worth noticing that compound **36** (homologue of **34**) demonstrated similar activities on the three *S. epidermidis* strains (RP62A, 40004, and 48155) at fourfold and eightfold the MIC. These results highlighted the potential role of distancing the carboxylic group from the phenyl ring concerning biofilms. The most active against three *S. epidermidis* strains was compound **37** at a concentration of eight-fold MIC, with a decreased amount of bacteria by 2.8 log_10_cfu/well (RP62A), 2.3 log_10_cfu/well (40004), and 1.8 log_10_cfu/well (48155) [52].

**Figure 16:**
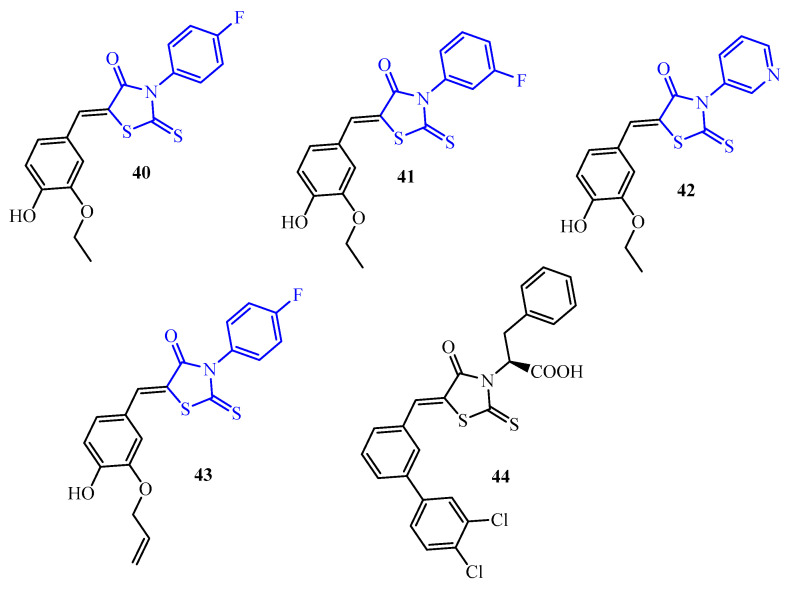
The structures of 3-arylrhodanines.

The next research concerned antibiofilm activities of the 3-arylrhodanines (**40**–**43**) (blue color in Figure 16). The evaluation of antibiofilm activities was carried out using a panel of Gram-positive (*S. aureus*, *S. epidermidis*, *E. faecalis*, *Enterococcus faecium*, and *Enterococcus gallinarum*) and Gram-negative strains (*E. coli* and *P. aeruginosa*) including resistant strains. The results of this study demonstrated that the 3-arylrhodanines **40**–**43** have potent antibiofilm activity against a wide range of Gram-positive bacteria. Compounds **40**–**43** exhibited good antibiofilm activity toward *Staphylococcus* spp. in the range of MBIC = 2.6–100 µM. Most active among them were compounds **40** and **43** with MBIC 2.6 µM against *S. aureus* ATCC 35556 and derivatives **40**, **41**, and **43** that inhibited biofilm growth at a concentration of 8.8 µM against methicillin-resistant *S. aureus* MRSA-47263.

However, taking into account all panels of Gram-positive strains, compound **42** showed the best antibiofilm potential. Its MBICs against *S. aureus* strains were in the range of 10.5 to 31.5 µM, against *S. epidermidis* strains from 12.5 to 59.5 µM, against *E. faecalis* from 8.8 to 21 µM, against *E. faecium* from 15.7 to 21 µM, and toward *E. gallinarum* 2392 MBIC = 17.7 µM [53].

In turn, only one Gram-positive strain in the panel of *E. faecium* F1181 was resistant to all tested rhodanine derivatives (MBIC ≥ 100 µM). None of the compounds **40**–**43** showed antibiofilm activity toward Gram-negative *E. coli* ATCC 25922 and *P. aeruginosa* PAO1 strains (MBIC ≥ 100 µM).

Additionally, the mechanism of action studies revealed that 3-arylrhodanine derivatives specifically inhibit the early stages of biofilm formation by preventing the adhesion of the bacteria to surfaces.

Rhodanine derivative **44** at four- and eight-fold its MIC (4 µM) significantly decreased *S. epidermidis* biofilm mass by more than 35% and 45%, respectively. In contrast, even at high concentrations, neither linezolid nor vancomycin significantly reduced biofilm formation [54].

**Figure 17:**
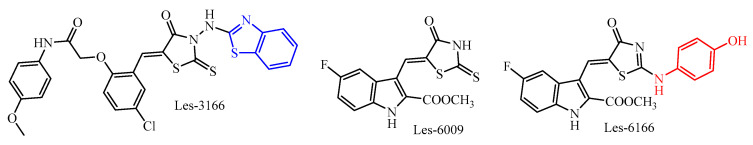
The structures of rhodanine derivatives with benzothiazole and indole moieties.

The rhodanine derivative (Les-3166) with benzothiazole moiety (blue color in Figure 17) induced significant degradation of the *P. aeruginosa* biofilm after 24-h exposure to all examined concentrations. The most pronounced antibacterial effects were observed at concentrations of 10, 50, and 100 µM, resulting in reductions of 37%, 34%, and 58%, respectively, compared to the control. In turn, the 5-(indol-3-yl)methylidenerhodanine derivative (Les-6009) exhibited the ability to inhibit the formation of *P. aeruginosa* biofilm at concentrations of 10, 50, and 100 µM, leading to decreases in biofilm biomass of 28%, 29%, and 45%, respectively, compared to the control. Notably, the replacement sulfanyl group at the second position of the thiazolidine ring by a 4-hydroxyphenylamine moiety (Les-6166) (marked red in Figure 17) did not induce any changes in biofilm eradication at any tested concentration [55].

### 4.4. Thiazolidine-2,4-Diones (TZD)

**Figure 18:**
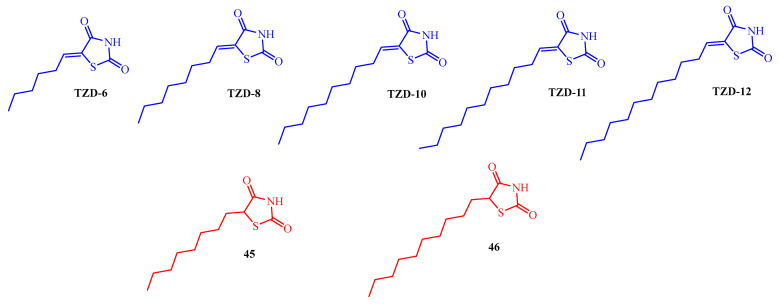
The structures of 5-alkylidene-TZD and some of their saturated analogues.

The interesting group of antibiofilm compounds was also the 5-alkylidenethiazolidine-2,4-diones (TZD-6, TZD-8, TZD-10, TZD-11, and TZD-12) and some of their saturated analogues (**45** and **46**) synthesized by Kagan and co-workers (blue and red colors in Figure 18, respectively) [56]. The compounds were tested for their antibiofilm activity against *Candida albicans*. The antibiofilm activity (as measured by the biofilm’s metabolic activity) was evaluated at sub-MICs. The MIC for TZD-10, TZD-11, TZD-12, and saturated analogues **45** and **46** was >200 μg/mL, whereas TZD-6 and TZD-8 showed activity against *C. albicans* at MIC = 50 μg/mL. A concentration of 100 μg/mL TZD-10 inhibited 61% of biofilm formation. On the other hand, TZD-6 and TZD-8 inhibited 50% and 43% of biofilm formation, respectively, at a concentration of 6.25 μg/mL. This effect depended on the length of the alkyl chain. In the case of TZD-12, when the alkyl chain consists of 12 carbons, antibiofilm activity is almost abolished.

The most effective TZD derivatives among this group, namely TZD-8, were selected for more detailed studies against *C. albicans* biofilm formation [57,58,59,60,61].

TZD-8 caused the detachment of preformed *C. albicans* biofilms and reduced metabolic activity in developed biofilms in a dose-dependent manner in comparison to the control. At concentrations of 1 μg/mL, 4 μg/mL, and 16 μg/mL, TZD-8 reduced metabolic activity by 21%, 61%, and 70%, respectively [57].

In the next studies, TZD-8 was tested on antibiofilm activity against co-species *C. albicans–Streptococcus mutans* [58]. No change in biofilm formation was observed when using 4 μg/mL of TZD-8. However, increasing concentrations of TZD-8 to 8 and 16 μg/mL revealed biofilm inhibition by 42% and 59%, respectively. Additionally, TZD-8 substantially reduces EPS production in a dose-dependent manner. Furthermore, TZD-8 induces a complex alteration in the symbiotic relationship between these species. The expression of *Streptococcal* genes associated with quorum sensing (QS) (*comDE* and *luxS*), EPS production (*gtfBCD* and *gbpB*), and genes related to protection against oxidative stress (*nox* and *sodA*) are notably upregulated by TZD-8. In contrast, fungal genes related to hyphae formation (*hwp1*), adhesion (*als3*), hydrophobicity (*csh1*), and oxidative stress response (*sod1*, *sod2*, and *cat1*) are downregulated in the presence of TZD-8. These results suggest that TZD-8 disrupts the symbiotic balance between *C. albicans* and *S. mutans* within the dual-species biofilm [58].

A sustained-release membrane (SRM) of TZD-8, which has the properties of an anti-quorum-sensing agent, displayed good potential against oral fungal (*C. albicans*) biofilms [60]. Moreover, SRM-TZD-8 revealed an advantageous effect on the formation of *Candida/Streptococcus* mixed biofilm on hydroxyapatite in a continuous flow model [59]. The utilization of a locally applied sustained-release drug delivery system for TZD-8 has the potential to influence dental polymicrobial biofilm, leading to observable clinical improvements and enhanced patient compliance. Additionally, it was reported that local sustained-release delivery systems with antibiofilm agent TZD-8 were used for the prevention of catheter-associated urinary tract infections. As a matrix that influences the release rate of an antibiofilm agent, ethylcellulose was used [61].

In addition to its antibiofilm activity against *C. albicans*, TZD-8 also showed activity against other microorganisms that form biofilms.

Lidor et al. reported that TZD-8 displayed an anti-quorum-sensing effect on *P. aeruginosa* biofilm potentially through the inhibition of LasI and the associated gene regulation. The *Pseudomonas* quorum-sensing signaling in the PAO1 strain exhibited a dose-responsive manner upon exposure to the TZD-8 molecule. The reduction became noticeable with the initial exposure to 2 μM and 20 μM concentrations of TZD-8. The inhibitor targeting *P. aeruginosa* quorum-sensing, particularly the TZD-8 compound, holds significant promise (confirmed by in vitro and in silico tests). Its effectiveness not only showcases potential therapeutic benefits but also validates the mechanistic approach employed in the discovery of inhibitors targeting LuxI-type acyl-homoserine lactone synthases [62].

Furthermore, the potential of TZD-8 as an antibiofilm agent has also been confirmed against *Cryptococcal* biofilm. The compound was tested by XTT reduction assay for biofilm metabolic activity. TZD-8 demonstrated reductions in biofilm metabolic activity of two *Cryptococcal* strains when treated with sub-inhibitory concentrations (1/4 and 1/16 MIC). The metabolic activity of *Cryptococcal neoformans* H-99 biofilm was reduced by 51.6% and 47.8% at concentrations of 3.125 and 0.78 μg/mL, respectively. On the other hand, the metabolic activity of *Cryptococcal gattii* R-272 biofilm was reduced to a lower extent by 34.8% and 26.5% at concentrations of 6.25 and 1.56 μg/mL, respectively [63].

In addition, TZD-8 and TZD-10 (Figure 15) showed activity against *Propionibacterium acnes* biofilms as quorum-sensing inhibitors [64]. Another homologue of 5-octanylidenethiazolidine-2,4-dione, TZD-6, displayed activity against *Pectobacterium carotovorum* biofilm. Based on TZD-6, an anti-biofilm polymer designed to coat the surfaces of corrugated cardboard was developed, effectively moderating bacterial biofilm formation on those surfaces. The incorporation of a novel thiazolidinedione derivative into acrylic emulsion polymers was evaluated through energy-dispersive X-ray spectrometry analysis, while surface topography on corrugated cardboard surfaces was observed and quantified. Biofilm growth was assessed using q-PCR, targeting the gene responsible for encoding 16s rRNA. The thorough analysis confirmed the consistent integration of the thiazolidinedione derivative TZD-6. Through q-PCR analysis, it was established that TZD-6 reduced biofilm growth by approximately 80% on the surfaces under examination [65].

Moreover, 5-alkylidene-TZD (TZD-8, TZD-10, TZD-11, and TZD-12 in Figure 15) possessed QS inhibitory activity with EC_50_ values in the range of 2.1 to 29.4 µM and 9.8 to 34.6 µM against *Vibrio harveyi* BB170 and *V. harveyi* MM32, respectively. They inhibited the AI-2-mediated bioluminescence of *V. harveyi* BB170 and *V. harveyi* MM32 [66]. The most effective QS inhibitor among them was TZD-10 with EC_50_ values of 2.1 µM and 9.8 µM in *Vibrio harveyi* BB170 and *V. harveyi* MM32, respectively. These outcomes demonstrated that the length of alkylidene chain at the fifth position of the thiazolidine ring affected QS inhibitory activity. TZD might act on the transcriptional regulatory LuxR of an AI-2-regulated QS system to interfere with the binding between LuxR and DNA. The saturated analogue of TZD-10, compound **46**, exhibited QS inhibitory activity with EC_50_ = 21.9 µM (for *V. harveyi* BB170) and EC_50_ = 37.9 µM (for *V. harveyi* MM32) [67].

**Figure 19:**
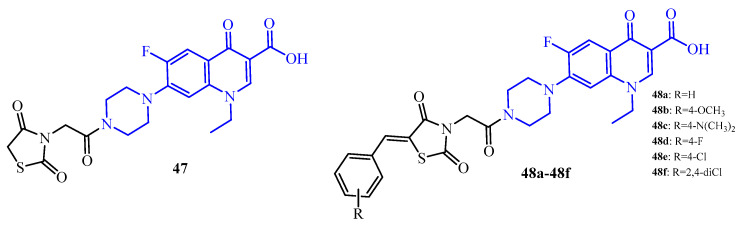
The structures of TZD-norfloxacin hybrids with antibiofilm activity.

Analysis of the antibiofilm activity of TZD-norfloxacin hybrids (**47**, **48a**–**48f**) showed that these derivatives were most active against *S. aureus* strain with MBEC in the range of 4.9 to 39.0 μg/mL (norfloxacin fragment marked blue color in Figure 19). The most active compounds among them were **47** (unsubstituted hybrid in thiazolidine ring), 4-fluorobenzylidene derivative (**48d**), and 2,4-dichlorobenzylidene derivative (**48f**) with MBEC = 4.9 μg/mL. Furthermore, compound **47** was most effective against *E. faecalis* (MBEC = 9.8 μg/mL) and *E. coli* (MBEC = 39.0 μg/mL). Compounds **48b**, **48c**, and **48f** proved to be the most effective against the Gram-negative strain of *P. aeruginosa* with MBEC = 78.1 μg/mL. On the other hand, TZD-norfloxacin hybrids (**47**, **48a**–**48f**) showed poor activity against fungal strains (*C. albicans* and *C. parapsilosis*). The values of their MBEC were in the range of 156.2 to 312.5 μg/mL for *C. albicans* and 156.2 to 2500 μg/mL for *C. parapsilosis*, except **48b** (MBEC = 78.1 μg/mL) [68].

**Figure 20:**
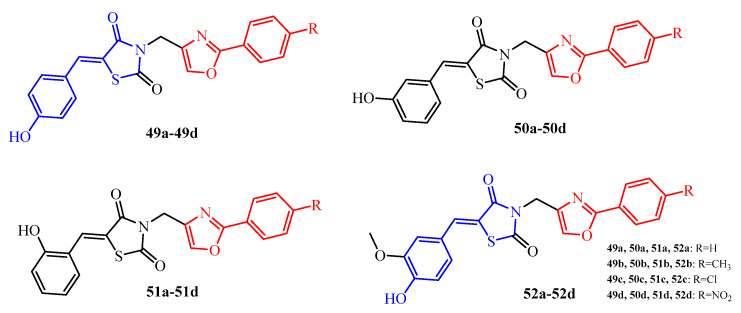
The structures of TZD-oxazole hybrids with antibiofilm activity.

The replacement of the norfloxacin scaffold by 2-aryl-1,3-oxadiazole moieties (**49a**–**52d**) (red color in Figure 20) practically abolished antibiofilm activity against the *S. aureus*, *E. faecalis*, *E. coli*, and *P. aeruginosa* strains with MBEC in the range of 156 to >625 μg/mL, whereas activity against *C. albicans* biofilm was more effective than the activity of the TZD-norfloxacin hybrids (MBEC 39–156 vs. 156.2–312.5 μg/mL) [69].

**Figure 21:**
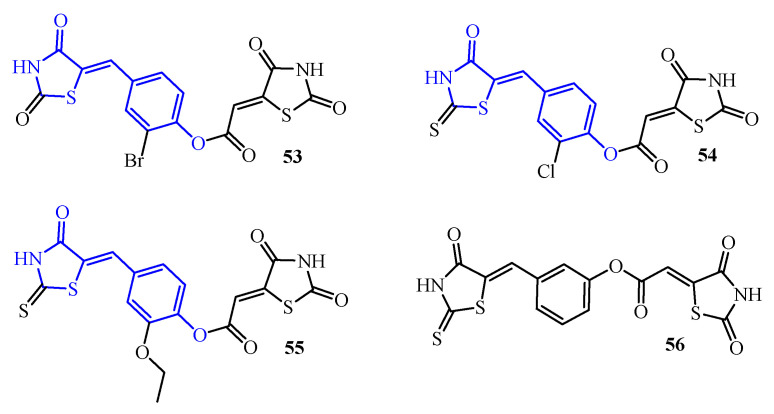
The structures of TZD-thiazolidinone hybrids.

The 5-(4-hydroxybenzylidene)thiazolidin-4-one motif (blue color in Figure 20) was also observed in derivatives (**53**–**56**) (marked blue in Figure 21). These compounds showed biofilm-forming inhibition of reference species (*Haemophilus parainfluenzae* ATCC 33392, *Haemophilus parainfluenzae* ATCC 51505, and *Haemophilus influenzae* ATCC 10211) with MBIC = 250 μg/mL for **53**, **54**, and **56**. Compound **55** exhibited activity against all reference strains with an MBIC value of 500 μg/mL. Moreover, compounds **53**, **55**, and **56** demonstrated activity against clinical isolate *H. parainfluenzae* 201 (MBIC = 125 μg/mL). Among them, the most active was compound **54**, which inhibited the biofilm forming of *H. parainfluenzae* 201 with MBIC = 62.5 μg/mL [70]. In addition, tested compounds (**53**–**56**) showed good anti-adhesive properties. The ability of the tested compounds to inhibit the initial phase of biofilm formation was contingent upon both the specific compound and its concentration. Moreover, the anti-adhesive properties demonstrated a reversible nature when bacteria were subjected to prolonged incubation in the presence of lower compound concentrations. This suggests that the inhibitory impact diminishes over time under reduced compound concentrations, highlighting the dynamic characteristics of the anti-adhesive properties during extended exposure periods.

The anti-adhesive properties of tested compounds were assessed after 1 h incubation in the conditions and by a method such as a biofilm. The assay was carried out according to the biofilm detection procedure. After 1 h of incubation, the cultures from microplates were removed; the wells were rinsed and, after drying, stained with 0.1% crystal violet for 10 min. After washing off the excess dye, the microwells were poured for 15 min with ethanol. Absorbance values were obtained using a 570 nm wavelength spectrometer (OD570) [70].

Among the compounds examined, namely **53**–**56**, those exhibiting the most pronounced anti-adhesive properties were characterized as derivatives of (2,4-dioxothiazolidin-5-ylidene)acetic acid, featuring a double bond in the fifth position of the TZD ring. Notably, these compounds predominantly incorporated a rhodanine ring into their structure, as observed in compounds **54**–**56**.

Interestingly, the substituent located in the second position of the phenyl ring did not appear to exert a significant influence on the anti-adhesive properties. Furthermore, the isomerization within the phenyl ring, as evidenced by the meta isomer compound **56**, seemed to have a negligible impact on these properties. This structural analysis provides insights into the specific features associated with the compounds that contribute to their heightened anti-adhesive effects in the context of biofilm formation.

The ability of compounds to affect microbial adherence as the first and key stage of biofilm formation may be an important criterion for the design of the substances with improved antibiofilm activity.

**Figure 22:**
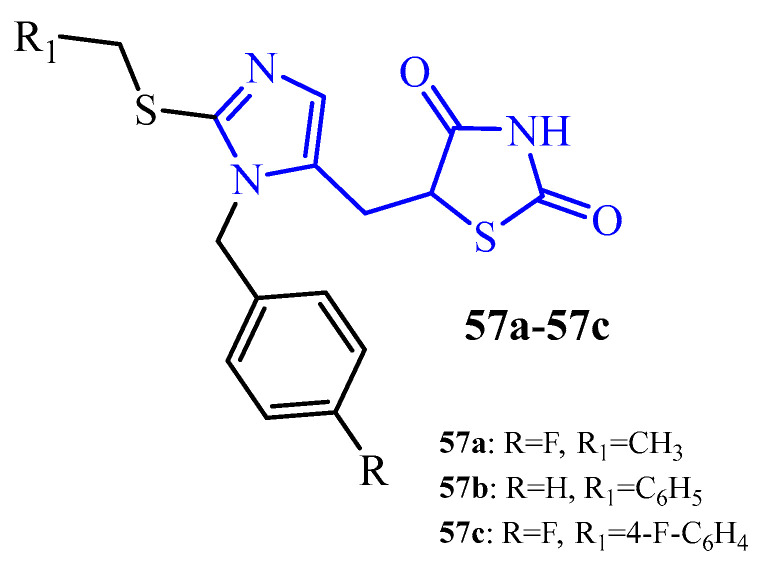
The structures of TZD-imidazole hybrids.

As reported by Shakour et al., TZD-imidazole hybrids (**57a**–**57c**) (blue color in Figure 22) showed a biofilm reduction of 87.94% (**57a**) for the *P. aeruginosa* PAO1 strain, as well as of 30.53% (**57b**) and 44.65% (**57c**) for the *P. aeruginosa* 1074 and *P. aeruginosa* 1707 strains, respectively. It is worth noticing that tested compounds did not show any toxicity against human dermal fibroblasts and 4T1 cells (viability higher than 90%) [71].

## 5. Key Structural Features of Thiazolidine-4-Ones Responsible for Antibiofilm Activity

**Figure 23:**
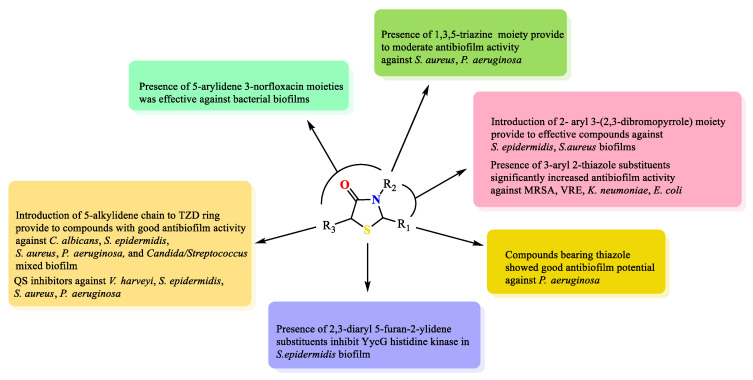
Key structural features for thiazolidine-4-ones as antibiofilm agents. Yellow color—substitution at the position 2; green color—substitutions at the position 3; beige color—substitutions at the position 5; pink color—substitutions at the positions 2 and 3; cyan color—substitutions at the position 3 and 5; blue color—substitutions at the positions 2, 3 and 5 of thiazolidine ring.

Based on the description provided in this review and on a thorough critical analysis of the scientific information presented herein, the pivotal structural features influencing the antibiofilm activity of thiazolidin-4-one derivatives can be identified. The important structural elements are included in Figure 23.

Introduction of the 5-alkylidene chain to the TZD ring provides compounds with good antibiofilm activity against *C. albicans*, *S. epidermidis*, *S. aureus*, *P. aeruginosa*, and *Candida/Streptococcus* mixed biofilm. Moreover, some of them inhibited QS signals against *V. harveyi*, *S. epidermidis*, *S. aureus*, and *P. aeruginosa*.

The presence of arylidene and norfloxacin moieties in positions 5 and 3 of the thiazolidine ring, respectively, was effective against bacterial biofilms but showed a weak effect on fungal biofilm formation.

On the other hand, the presence of the 1,3,5-triazine moiety in the third position in thiazolidine provides moderate antibiofilm activity against *S. aureus* and *P. aeruginosa*.

Introduction of the 2-aryl 3-(2,3-dibromopyrrole) moiety provides effective compounds against *S. epidermidis* and *S. aureus* biofilms. The presence of 3-aryl 2-thiazole substituents significantly increased antibiofilm activity against MRSA, VRE, *K. neumoniae*, and *E. coli*.

Compounds bearing thiazole in position 2 of thiazolidine showed good antibiofilm potential against *P. aeruginosa.*

The presence of 2,3-diaryl 5-furan-2-ylidene substituents is important to inhibit YycG histidine kinase in *S. epidermidis* biofilm.

## 6. Challenges and Future Perspective

The rise of severe biofilm infections and their resistance to antimicrobial treatment pose significant challenges in the medical field. Over the last two decades, there has been rapid progress in the study of microbial biofilms, revealing the intricate nature of this phenomenon. Despite this progress, the persistence of infections linked to biofilms continues to pose a significant health crisis. Therefore, concerted efforts are essential to deepen our comprehension of the genetics, physiology, and dynamics of bacterial biofilms, particularly concerning chronic infections.

Further investigations should aim to pinpoint the genes responsible for each stage of biofilm development, including those critical for the initial transition from individual cells to aggregate forms. Additionally, understanding the mechanisms through which biofilms acquire antimicrobial resistance is crucial. Simultaneously, the quest for innovative antibiofilm agents targeting biofilm-specific bacterial components is imperative. Thiazolidin-4-ones completely fit into this assumption.

The future directions of small molecule development as antibiofilm agents include thiazolidin-4-ones and involve using innovative multipronged antibiofilm therapeutic strategies, incorporating nanoengineering (antibiofilm nanoparticles), antibiofilm surface coating, or antibiofilm microneedles. The latter can effectively penetrate the EPS barrier of biofilms, offering an efficient drug delivery system.

## 7. Conclusions

In conclusion, this paper provides an overview of the antibiofilm properties of various thiazolidin-4-ones, which have been discovered during the last two decades. There are tests against biofilm formation, as well as more in-depth research related to finding the mechanisms of action of thiazolidin-4-ones. Some of them have promising anti-adhesive properties, reduce EPS production, or inhibit quorum-sensing signaling. Compound **3b** showed significant antibiofilm activity against the MRSA and VRE strains (BIC values of 2.22 µg/mL and 3.05 µg/mL, respectively), as well as significant activity against *K. pneumoniae* and *E. coli* biofilms with BIC values of 3.25 µg/mL and 2.03 µg/mL, respectively. Compounds **20** and **24** exhibited bactericidal activity against six strong biofilm-forming staphylococcal clinical isolates. Thiazolidine-4-ones (**32a**–**32h**) demonstrated equal *S. epidermidis* antibiofilm activity compared to the standard vancomycin (MIC = 3.125 µg/mL). Moreover, **32b** and **32c** exhibited high effective inhibition of *S. aureus* biofilm at a concentration of 0.78 µg/mL.

In turn, some thiazolidine-4-ones showed antibiofilm activity against *S. epidermidis* biofilm as a YycG histidine kinase inhibitor. The compound TZD-8 demonstrated broad-spectrum antibiofilm activity against *C. albicans*, *S. epidermidis*, and *P. aeruginosa*, as well as mixed Candida/Bacteria biofilm formation. Furthermore, TZD-8 can be used in clinical aspects for the prevention of catheter-associated urinary tract infections or can affect dental polymicrobial biofilm. Its homologue (TZD-10) was the most effective QS inhibitor in *V. harveyi* BB170 at a micromolar concentration (EC_50_ = 2.1 µM).

Analyzing the results, it can be concluded that the group of thiazolidin-4-one derivatives have great potential as antibiofilm agents and can act as inhibitors of QS signaling, reduce EPS production, or display anti-adhesive properties. Therefore, this review may be useful for the further development of new molecules based on a thiazolidin-4-one core as potential bioactive agents to combat biofilm formation.

## Data Availability

Data are contained within the article.
